# Mucorales PCR in blood as an early marker of invasive gastrointestinal mucormycosis might decrease the delay in treatment: A case report

**DOI:** 10.1016/j.mmcr.2022.12.001

**Published:** 2022-12-15

**Authors:** Robina Aerts, Greet De Vlieger, Yves Debaveye, Halit Topal, Gert De Hertogh, Katrien Lagrou, Johan Maertens

**Affiliations:** a Department of Internal Medicine, University Hospitals Leuven, Leuven, Belgium; b Department of Microbiology, Immunology and Transplantation, KU Leuven, Leuven, Belgium; c Intensive Care Medicine, University Hospitals Leuven, Leuven, Belgium; d Department of Cellular and Molecular Medicine, Laboratory of Intensive Care Medicine, KU Leuven, Leuven, Belgium; e Department of Visceral Surgery, University Hospitals Leuven, Leuven, Belgium; f Department of Pathology, University Hospitals Leuven, Leuven, Belgium; g Department of Laboratory Medicine, University Hospitals Leuven, Leuven, Belgium; h Department of Hematology, University Hospitals Leuven, Leuven, Belgium

**Keywords:** Mucorales PCR, Invasive mould infections, Mucormycosis, Critically ill patient, Opportunistic infections

## Abstract

We describe the fatal case of a patient with gastric perforation due to ischemia and necrosis of the stomach secondary to generalized vascular thrombosis following allogeneic hematopoietic cell transplantation. Histopathological examination of the resected stomach, spleen and omentum unexpectedly showed fungal hyphae suggestive of invasive mucormycosis. We retrospectively performed Mucorales PCR (MucorGenius®, PathoNostics, Maastricht, The Netherlands) in blood and tissue samples of this patient. The PCR was positive 16 days before time of death and 9 days before abdominal pain.

## 1 Introduction

Mucormycosis is a difficult to diagnose rare disease with high morbidity and mortality [1]. Rhino-orbito-cerebral and pulmonary mucormycosis are the most common clinical presentations. Invasion into other organs, such as the gastrointestinal system, has been less frequently reported and symptoms are typically non-specific [2,3]. Suspected mucormycosis urges prompt intervention because of the rapidly progressive and destructive nature of the infection. Delayed initiation of a complex multidisciplinary approach is associated with increased mortality. However, the combination of being a very rare infectious disease and non-specific symptoms hamper the diagnosis often leading to a late treatment initiation [4].

Recently, several PCR assays for Mucorales DNA detection have been developed. These assays show good sensitivity and specificity when used on blood samples [3,5,6] and often precede the final diagnosis by several days to weeks [7]. The MucorGenius® (PathoNostics, Maastricht, The Netherlands) uses a real-time PCR to detect DNA from *Rhizopus* spp., *Mucor* spp., *Lichtheimia* spp., *Cunninghamella* spp., and *Rhizomucor* spp. By targeting the 28S rRNA gene and an M13 bacteriophage as internal control. Little is known about the implementation of this PCR assay in blood from at-risk patients with gastrointestinal symptoms. Herein we report on the potential added value of PCR technology in a fatal case of proven abdominal mucormycosis following allogeneic hematopoietic cell transplantation.

## 2 Case presentation

In November 2021, a 37-year-old male patient with a medical history of well-controlled inflammatory bowel disease under infliximab was diagnosed with mixed phenotype acute leukaemia (MPAL). Infliximab was stopped at the time of diagnosis. Following remission-induction polychemotherapy, which resulted in a complete morphological and immunophenotypical remission of the bone marrow, and consolidation chemotherapy, he underwent a myeloablative (based on fludarabine and total body irradiation) haplo-identical allogeneic hematopoietic cell transplantation in March 2022 (Day 0). On day +69 posttransplant, he was admitted with histopathology-proven acute lower intestinal graft-versus-host disease (GvHD), MAGIC grade IV. Because of deteriorating renal function, tacrolimus had been switched to methylprednisolone (32 mg/day) two weeks prior to hospitalisation. After pathological confirmation of acute intestinal GvHD, the dose of methylprednisolone was increased to 1 mg per kg body weight twice daily intravenously. Nevertheless, there was a further deterioration of the clinical status, with a productive and painful cough, diarrhea and general weakness, combined with an increase in the inflammatory parameters for which empirical treatment with intravenous cefepime (2g TID) was initiated. Two sets of blood cultures yielded *Enterococcus faecium* for which vancomycin (continuous infusion) was added.

**Fig. 1:**
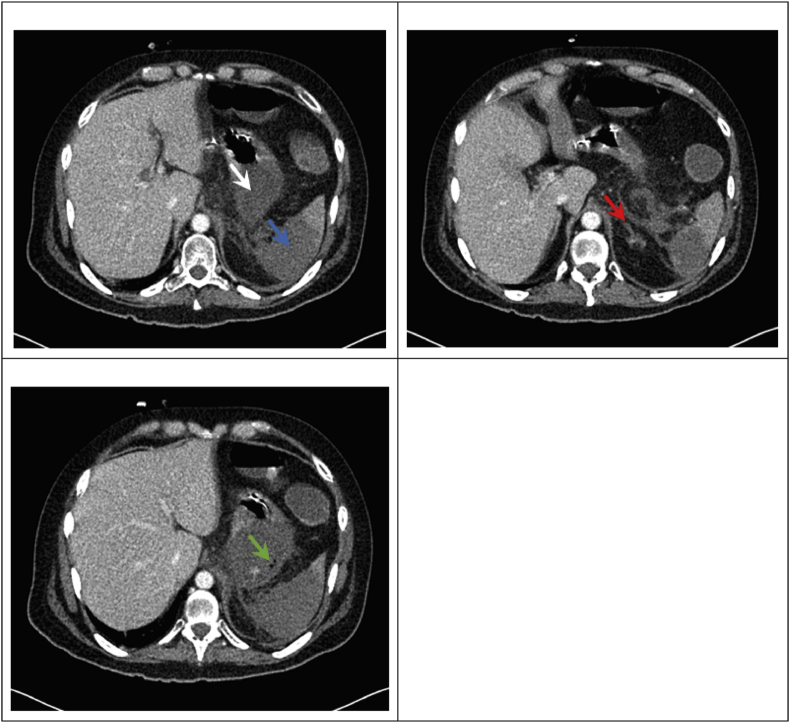
CT images during the venous phase. Subtotal infarct zone of the spleen (blue) with contrast enhancement only in an anterior segment of the spleen. Also partial infarction of the left adrenal gland (red), the gastric fundus and proximal gastric corpus (white) with adjacent free air bubbles (green) suggestive of gastric perforation. (For interpretation of the references to colour in this figure legend, the reader is referred to the Web version of this article.)

Gradually the patient became neutropenic and developed fever while receiving broad-spectrum antibiotic treatment; blood cultures were repeatedly negative. High-resolution chest CT scan showed no features suggestive of an opportunistic pulmonary infection (Day 74) and an abdominal CT scan showed small bowel dilatation with wall thickening suggestive of GvHD (Day 75). There were no signs of sinusitis and no cutaneous lesion was found. Selected blood results are summarized in Table 1. As the clinical presentation suggested persistent GvHD, ruxolitinib (10 mg BID) was added while corticosteroids were gradually tapered. The following day, the patient developed progressive pan-abdominal pain. A repeat abdominal CT scan showed thrombosis of the splenic artery with infarction of the spleen and the left adrenal gland as well as suspicion of perforation of the stomach (Fig. 1), after which the patient was admitted to the intensive care unit (ICU) (Day 85).

**Table 1:** Lab results. NP = not performed.

Lab results	Normal values	At hospital admission	At ICU admission	One day before death
Hb (g/dL)	14.0–18.0	9.8	7.9	8.1
WBC (/μL)	4000 - 10 000	6190	960	1420
Neutrophils (/μL)	2500–7800	4900	800	1200
Thrombocytes (/μL)	150 000–450 0000	40 000	16 000	41 000
CRP (mg/L)	≤0.5	85.3	438.7	314.9
Bilirubin (mg/dL)	≤1.18	0.50	4.92	13.09
AST (U/L)	≤37	12	69	1168
Ferritin (μg/L)	30–400	NP	NP	59 085
Galactomannan (OD)	<0.5	0.2	0.0	0.0
Beta-D-glucan (pg/mL)	≤7.0	NP	<2.9	NP
MucorGenius®	Cq < 40 considered positive	Negative	Positive Ct 25.9	Positive Ct 24.2

Laparoscopic exploration of the abdomen confirmed gastric ischemia in the proximal part without a visible perforation (Day 85). After intraoperative interdisciplinary discussion, we decided not to perform a gastrectomy, given the severe acute GvHD and the high operative risk. Only the proximal part of the stomach was affected and, because of the abundant blood supply of the stomach, it was expected the ischemic zones would recover. Moreover, a total gastrectomy with RY oesophagoenterostomy anastomosis would be needed if resection was performed. An additional transthoracic echocardiography showed no evidence of thrombotic events. Heparin-induced thrombocytopenia (HIT) was excluded and thrombotic microangiopathy was deemed unlikely due to the absence of schistocytes. Blood cultures and cultures from other relevant sites (urine, abdominal cultures) remained negative as well as serial serum galactomannan and serum beta-D glucan detection testing.

**Fig. 2:**
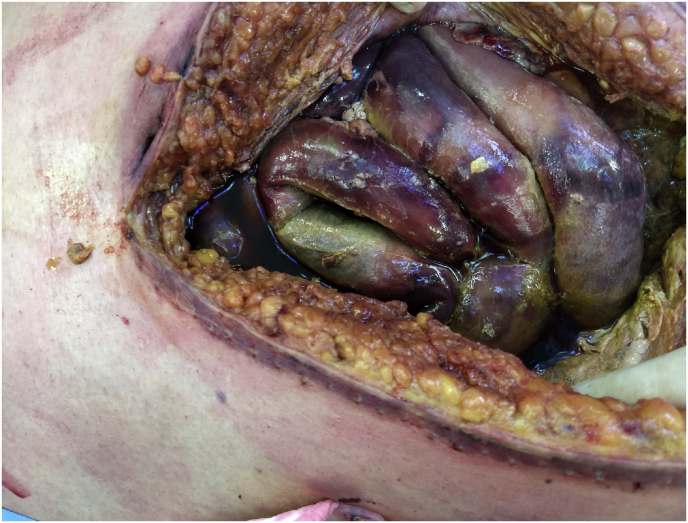
Midline laparotomy shows extensive ischemia/necrosis of small intestine, including enteroenterostomy and jejunostomy.

**Fig. 3:**
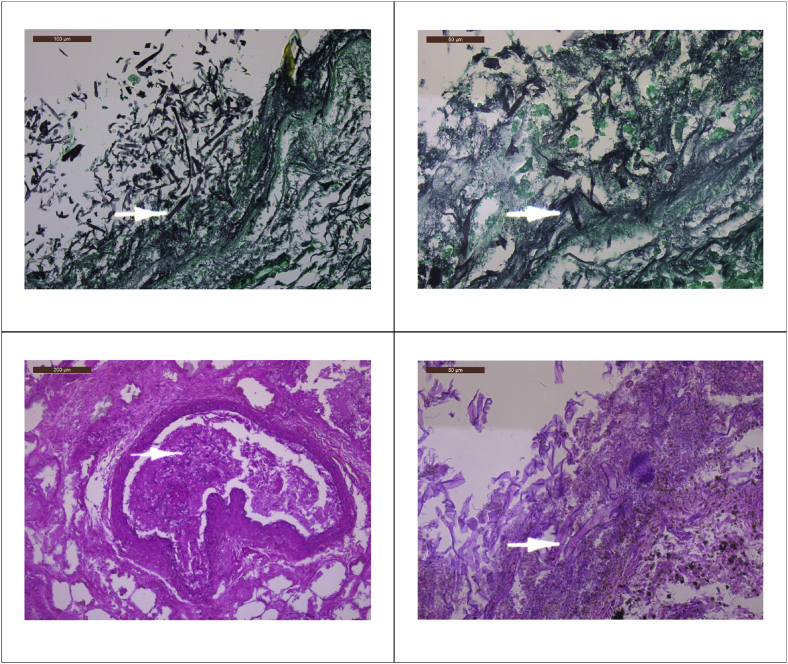
Pathological examination of the resected stomach showing haemorrhagic necrosis and thrombosis with an abundant presence of minimally septated broad, ribbon-like hyphae invading blood vessels, morphologically consistent with invasive mucormycosis.

Because of further evolution to multiple organ failure with progressive liver dysfunction and pleuritic effusion, source control with a total gastrectomy and splenectomy was performed (Day 89). Early second look surgery was anticipated because of the visualisation of ischemic areas of the small intestine and colon. Meanwhile the patient's condition further rapidly deteriorated with progressive liver failure, lactic acidosis and need for mechanical ventilation. A new explorative laparotomy showed necrosis of the entire small intestine (Day 91) (Fig. 2); the patient succumbed the next day (Day 92). Pathological examination of the resected stomach, spleen and omentum showed haemorrhagic necrosis and thrombosis with an abundant presence of minimally septated broad, ribbon-like hyphae invading blood vessels, morphologically consistent with invasive mucormycosis (Fig. 3). The patient has never received antifungal therapy covering Mucorales species.

**Fig. 4:**
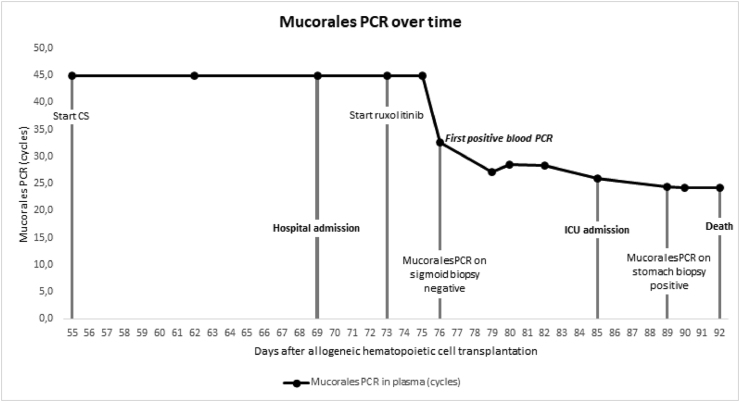
Mucorales PCR in plasma over time; CS = corticosteroids.

Following the unexpected post-mortem diagnosis of proven abdominal mould disease, presumably mucormycosis, we retrospectively tested the commercially available Mucorales PCR (MucorGenius®, PathoNostics, Maastricht, The Netherlands) on resected tissue specimens and stored plasma samples (banked left-over serum samples used for serial galactomannan screening) (Fig. 2). We extended our search period back in time until the samples became negative. From time of death backwards until the first negative sample, 8 plasma samples were available in our biobank and were tested. Mucorales PCR was positive on the tissue specimen of the resected stomach as well as in plasma up to 16 days before death and 9 days before the development of progressive abdominal pain and before ICU admission (Fig. 4). Mucorales PCR on the sigmoid biopsy (Day 76) showing GvHD was negative. During the course of clinical decline of the patient, quantitative Ct values of the Mucorales PCR decreased as well, suggesting increasing fungal load.

## 3 Discussion

Prolonged, profound neutropenic patients and allogeneic hematopoietic cell transplant (HCT) recipients are at increased risk of invasive mucormycosis (IM). Despite the availability of new antifungal drugs and substantial advances in diagnostic techniques, the management of IM remains complex. Poorest prognosis is observed in patients with hematological malignancies and in HCT recipients [1,6]. Gastrointestinal involvement of IM is rare and mortality is high [8]. Biopsy is often required to confirm this difficult to find diagnosis. Circulating Mucorales DNA in blood can be a sensitive biomarker often preceding the diagnosis made by conventional methods [7,9]. A handful of cases of gastric or oesophageal mucormycosis in which a Mucorales PCR was performed on tissue have been reported in the past (8,9), but only little is known about the implementation of this PCR assay in blood (plasma or serum) from at-risk patients with gastrointestinal symptoms.

Retrospective analysis of the Mucorales PCR in blood of this patient post-mortem diagnosed with IM was positive more than two weeks before death and more than a week before transfer to ICU. Our case-report suggests that the test in blood could be of value in the differential diagnosis of at-risk immunocompromised patients with bowel obstruction, abdominal ischemia or other atypical clinical presentations. We have found only one other case of gastrointestinal mucormycosis where PCR was performed in blood [10]. There is an additional case of disseminated mucormycosis, diagnosed post-mortem in gastric fluid, in which PCR on serum also was positive [11].

Our case report provides serial plasma Mucorales PCR results in an untreated patient with abdominal mucormycosis, showing early positivity. Assessing Mucorales PCR in blood can be a non-invasive aid to early diagnose and treat this devastating disease in critically ill at-risk patients.

## Ethical form

Informed consent was obtained from the patient's family for publication of this case report and any accompanying images.

## Funding source

Funding for additional PCR testing was possible through an earlier grant from 10.13039/100004319Pfizer.

## Consent

Written informed consent was obtained from the patient or legal guardian(s) for publication of this case report and accompanying images. A copy of the written consent is available for review by the Editor-in-Chief of this journal on request.

## Declaration of competing interest

The authors declare that there are no conflicts of interest.
